# Mycotoxin production by three different toxigenic fungi genera on formulated abalone feed and the effect of an aquatic environment on fumonisins

**DOI:** 10.1080/21501203.2019.1604575

**Published:** 2019-04-14

**Authors:** Mariska Riana Greeff-Laubscher, Ilze Beukes, Gert Johannes Marais, Karin Jacobs

**Affiliations:** a Department of Microbiology, Stellenbosch University, Stellenbosch, South Africa; b Department of Plant Pathology, Stellenbosch University, Stellenbosch, South Africa; c Department of Plant Sciences, University of the Free State, Bloemfontein South Africa

**Keywords:** Mycotoxins, Fusarium, Aspergillus, Penicillium, abalone, feed

## Abstract

Mycotoxins are toxic secondary metabolites produced by various filamentous fungi, of which *Fusarium, Aspergillus* and *Penicillium* are the three main genera. *Fusarium verticillioides* is one of the most dominant toxigenic fungal species, associated with fumonisin contamination in grain-based feeds, such as compound abalone feed. Mycotoxin production is influenced by temperature and available nutrients. In this study the aims were: to determine if abalone feed as growth substrate favours mycotoxin production for toxigenic fungi; to determine the most effective temperature for fumonisin production by *F. verticillioides* on abalone feed; and to assess the effect of the aquatic environment on fumonisin-contaminated abalone feed. A total of 93 fungal isolates were inoculated onto abalone feed, including species belonging to the genera *Fusarium, Aspergillus* and *Penicillium*. Feed inoculated with *F. verticillioides* were incubated at two different temperatures and fumonisin-contaminated feed was submerged into seawater for 24 h. Results showed that mycotoxins were produced when abalone feed was inoculated with toxigenic fungi, and that *F. verticillioides* produced higher concentrations of fumonisins at a lower temperature. Submerging fumonisin-contaminated feed in seawater showed that this toxin leached into the seawater, lowering the risk of fumonisins to be consumed by abalone.

## Introduction

Mycotoxins are low-weight molecular secondary metabolites mainly produced in the mycelial structures of certain filamentous fungi, during fungal growth (D’Mello and Macdonald 1997; Placinta et al. 1999; Leslie and Summerell 2006; Bhat et al. 2010). These secondary metabolites are toxic to humans and animals when consumed and can even be carcinogenic, neurotoxic, nephrotoxic or immunosuppressive – especially when chronic exposure occurs (Gelderblom et al. 1988; Hussein and Brasel 2001; Lazicka and Orzechowski 2010; Feijó Corrêa et al. 2018). The most common type of exposure in humans and animals is by consumption of contaminated feedstuffs or foods (Rheeder et al. 1992; Bhat et al. 2010; Mwanza 2011). Fungi known to produce mycotoxins are referred to as toxigenic fungi. This group is dominated by three genera, namely *Aspergillus, Penicillium* and *Fusarium*, and to a lesser extent other genera that include *Alternaria, Claviceps* and *Stachybotrys* (Bennett and Klich 2003; Wambacq et al. 2016). *Fusarium* species represent a number of plant pathogens, and are responsible for infection before and during harvesting, while *Penicillium* and *Aspergillus* more commonly colonise commodities and foods during drying and storage (Sweeney and Dobson 1998; Placinta et al. 1999; Miller 2008; Osibona et al. 2018).

*Aspergillus* species are responsible for the production of aflatoxins. Aflatoxins are made up of several toxic compounds, of which B1 (AFB_1_) is the most abundantly produced. The optimum growth rate of these species and the optimum temperature for aflatoxin production are almost identical, with 10–43°C and 12–40°C, respectively (Sweeney and Dobson 1998). Severe liver and brain damage has been linked to aflatoxin-contaminated feed, along with other clinical symptoms, such as skin lesions, yellowing of the body surface and eye cataracts (El-Sayed and Khalil 2009; Baldissera et al. 2018). The susceptibility to aflatoxins varies between species. While warm water fish such as channel catfish are less susceptible, rainbow trout are highly susceptible (Lee et al. 1968; Schoenhard et al. 1981; Manning 2005; Santacroce et al. 2008).

Ochratoxins are produced by storage fungi, such as certain species of *Penicillium* and *Aspergillus*, with ochratoxin A (OTA) being the most common. Production of OTA by species like *P. verrucosum* takes place at more temperate climates (4–31°C), while production of OTA by *A. ochraceaus* takes place at higher temperatures up to 37°C (Moss 2002a; Manning 2010). Not only does the temperature differ between these two species for optimal OTA production, but they also have different substrate requirements. *Aspergillus ochraceus* produce OTA optimally when grown on oil seeds such as peanuts and soybeans while, *P. verrucosum* produce OTA optimally when grown on cereal grains like maize, barley and wheat (Klich 2007; Manning 2010). Ochratoxin A caused necrosis of kidney tubular and liver cells, along with a reduction in weight gain in rainbow trout (Doster et al. 1974; Manning 2005).

Mycotoxins produced by *Fusarium* are widespread contaminants of animal feed, and have been shown to cause biological side effects even when present at low concentrations (Marasas et al. 1980; Gelderblom et al. 1988; Rheeder et al. 1992; Moss 2002b; Tuan et al. 2003; Adeyemo et al. 2018). Trichothecenes and fumonisins are the major mycotoxin groups associated with *Fusarium* spp. (Sweeney and Dobson 1998; Hussein and Brasel 2001; Moss 2002b; Desjardins and Proctor 2007; Miller 2008). Optimal temperature for mycotoxin production (25 ± 1°C) falls within the optimal growth temperature for *Fusarium* spp. Fumonisins are mainly produced by *F. verticillioides* and are heat stable, water soluble and consist of four series, the B series being the most abundant naturally occurring group (Musser et al. 1996; Seo and Lee 1999; Yildirim et al. 2000; Rheeder et al. 2002). The metabolic disruptions caused by fumonisins in fish can lead to cellular deregulation, cell death and a decrease in weight gain (Goel et al. 1994; Yildirim et al. 2000; Tuan et al. 2003; Adeyemo et al. 2018). Reduction in weight gain of any cultured animals has a negative impact on farm production. Fumonisins are water soluble (Alberts et al. 1990), and it is possible that when aquatic animals are fed under normal conditions they will consume very little to no fumonisins, especially when feed is not consumed immediately, but left in the water for the animals to eat on demand. Associated risks of mycotoxins in abalone are unknown.

In South Africa, the abalone industry makes use of commercially produced feed, mainly produced from a protein source and locally sourced grains. This is a favourable medium for fungal growth (Greeff-Laubscher et al. 2018). The feeding ratio for these cultured abalone is ±1 g feed/952.38 mL. However, feed is not always consumed immediately and can stay in the water for up to 32 h before it is consumed or flushed by the water system. Most farms have their water completely replaced every two hours, which can potentially remove contaminants from the system.

Mycotoxin production is influenced by several factors, including environmental conditions, fungicides, fertilisers and the interactions between toxigenic fungal species. In addition, strain specificity, variation and instability can also affect mycotoxin production (Luchese and Harrigan 1993; Placinta et al. 1999; Brzonkalik et al. 2011). The first aim of this study was to determine the ability of previously isolated and identified toxigenic fungal species to produce mycotoxins on abalone feed, under storage conditions. The second aim was to determine the impact of two different average temperatures – 16°C and 26°C (representing winter and summer temperatures in South Africa along the coastline) on fumonisin production by *F. verticillioides*, previously isolated from abalone feed. The final objective was to assess the effect of the aquatic environment on fumonisin contaminated abalone feed. This is important to determine the fumonisin concentration in feed at the time of consumption. Understanding the changes in mycotoxin levels when feed contaminated with fumonisins is exposed to water, can assist in future risk assessments to determine allowable limits in compound feed intended for aquatic animals.

## Materials and methods

### Sample preparation

#### Different toxigenic genera

Twenty one (21) *Fusarium*, 36 *Aspergillus* and 36 *Penicillium* strains previously isolated from abalone feed, were inoculated onto PDA (Potato dextrose agar), MEA (Malt extract agar) and CYA (Czapek yeast agar) respectively, from 15% glycerol stocks that were kept at 4°C. Cultures were incubated at 26 ± 1°C for 10 days. *Fusarium verticillioides* MRC0826 was included to serve as a positive control for fumonisin production (Rheeder et al. 2002). *Aspergillus flavus* MRC3951 (Thembo et al. 2010) was included as a positive control for the production of aflatoxins and *P. viridicatum* MRC0356 as an ochratoxin producing strain. Negative controls were set up containing sterile feed and water with no fungal inoculum, and natural controls containing sterile water and non-sterile feed, in which no inoculum was included.

#### Different temperatures and water submerging

Three different isolates (MRL117, MRL124, MRL336), all identified as *Fusarium verticillioides*, were used in this part of the study. The selected isolates produced the highest amounts of fumonisins on abalone feed as growth substrate (previous experiment). Cultures were stored in 15% glycerol at 4°C. Cultures were first inoculated on water agar to confirm viability. Throughout the experiment cultures were kept on water agar. Seven days before inoculation, cultures were transferred onto Potato dextrose agar (PDA) and incubated at 26°C.

### Inoculation and incubation conditions

Erlenmeyer flasks were prepared with abalone feed (Production date 24/04/2015; Batch #114/15) and MilliQ water in a 1 g: 1.2 mL ratio (Table 1). Flasks were covered with cotton wool and tin foil, left overnight at room temperature and autoclaved the following day. Overnight incubation was done to allow the feed to absorb the water. Sterile flasks were aseptically inoculum with agar plugs (2 mm) from one of the tester strains. Negative controls with no inoculant were included as well as natural controls. Natural controls were represented by feed left to rot naturally, resulting in a natural fungal community rather than a pure culture colonising the feed. All flasks were incubated (See Table 1 for respective times and temperatures), followed by mycotoxin analyses.

**Table 1.** Inoculation and incubation information for three different experiments to achieve three different objectives.

	Feed (g): Water (mL)	Genera inoculated	Incubation period (days)	Incubation temperature (°C)	Positive control strain
***Objective 1***	*25 ± 0.02: 30*	*Aspergillus spp*. *Penicillium spp*. *Fusarium spp.*	*42*	*26 ± 1*	*A. flavus MRC3951* *P. viridicatum MRC356* *F. verticillioides MRC826*
***Objective 2***	*25 ± 0.02: 30*	*Fusarium spp*	*70*	*16 ± 1* *26 ± 1*	*F. verticillioides MRC826*
***Objective 3***	*4.2 ± 0.01: 5*	*Fusarium spp*	*42*	*26 ± 1*	*F. verticillioides MRC826*

In preparation for water exposure, sufficient flasks were prepared to perform fumonisin extractions on feed before water exposure, and after 24 h of water exposure. Experiments were done in triplicate. After the incubation period, flasks were randomly divided into three groups with three flasks each. One group was labelled as “before water exposure” and the second group was labelled as “water exposure”. The same protocol was followed for both the control groups.

### Water exposure

Sterile plastic containers were prepared with 4 L autoclaved seawater and plastic pipes (5 mm thick) for aeration. Flasks labelled as “before water exposure” were set aside for fumonisin extractions and the contents of the flasks labelled as “water exposure” were added into the seawater directly from the Erlenmeyer flasks. Feed was left in the water for 24 h. Water samples were taken before feed was added (T0). Thereafter, water samples were taken 2 h (T1), 8 h (T2) and 24 h (T3) after the feed was added into the water. Water samples were taken with a sterile syringe and filtered through a Minisart (National Separations (Pty) Ltd., South Africa) 0.02 μm (regenerated cellulose) RC syringe filter, into a 2 mL screw neck amber glass vial and sent to the Central Analytical Facility (CAF), Stellenbosch University for fumonisin quantification, using liquid chromatography tandem mass spectrometry LC-MS/MS.

### Mycotoxin analyses

A toxin extraction protocol adapted from Szécsi et al. (2005) was used to extract the various mycotoxins. Extractions were done by adding 70% analytical grade methanol extraction buffer (4 mL: 1 g feed) into the flasks. Feed was crushed and stirred with a stainless steel spatula and then incubated on a shaker at 180 rpm, for 60 min at 25°C. Following incubation, 15 mL of the slurry was filtered through single layer cheesecloth into a 50 mL centrifuge tube. Tubes were centrifuged for 10 min at 4°C at 500 rpm. Thereafter, 2 mL supernatant was filtered into a 2 mL microcentrifuge tube through a Minisart (National Separations (Pty) Ltd., South Africa) 0.02 μm (regenerated cellulose) RC syringe filter. After overnight incubation at 4°C, samples were centrifuged for 10 min at 14,000 rpm. After centrifugation, 1800 μL supernatant was transferred to a 2 mL screw neck amber glass vial (Rose et al. 2013). The analytes, together with the freshly prepared standard dilution series of known concentration, were sent to the Central Analytical Facility (CAF), Stellenbosch University for quantification of fumonisins (FB1, FB2 and FB3), trichothecenes (DON, NIV, 15ADON, Fx) ZEA, aflatoxins (AFB1, AFG1, AFB2 and AFG2), and OTA, using liquid chromatography tandem mass spectrometry (LC-MS/MS). The analytical standards, used to prepare the standard dilution series, were obtained from Sigma- Aldrich (South Africa), and were all guaranteed >98% pure. See Table 2 for the concentrations measured.

**Table 2.** Mycotoxin measuring limits (mg/L) for this study.

Mycotoxins	Lowest measured concentration	Highest measured concentration
***FB_1_***	*0.01008*	*201.6*
***FB_2_***	*0.0101*	*202*
***FB_3_***	*0.00104*	*20.8*
***DON***	*0.0064*	*100*
***NIV***	*0.0064*	*100*
***15ADON***	*0.0064*	*100*
***Fx***	*0.0064*	*100*
***ZEA***	*0.0128*	*200*
***AFB_1_***	*1.5333* × *10^–5^*	*3.8333* × *10^–1^*
***AFB_2_***	*4.4267* × *10^–6^*	*1.1066* × *10^–1^*
***AFG_1_***	*1.5333* × *10^–5^*	*3.8333* × *10^–1^*
***AFG_2_***	*4.4267* × *10^–6^*	*1.1066* × *10^–1^*
***OTA***	*0.016196*	*0.259146*

Mycotoxins were detected in the sterile control samples. These mycotoxins were not produced under laboratory conditions and were present in the feed when collected from the supplier. Values from analysis of the sterile control samples were considered as being the mycotoxin concentration of feed without any fungal growth. These values served as a baseline and were deducted from the mycotoxin values measured in inoculated samples to achieve a true value of the mycotoxin concentration produced by a specific isolate. Provision was made for potential sample carry-over, by deducting 0.05% (% specified by the auto-sampler) from values following readings greater than 0.5 mg/L. Sample carry-over occurs when the liquid of a previous sample elutes upon subsequent samples due to the chemical/physical characteristics of the sample, analysis system or both. This takes place especially when very high concentrations of the measuring substance, in this case the mycotoxins, are present in a single sample. As a result, a subsequent sample is contaminated and does not reflect the true concentration of the specific sample. Samples that were exposed to water for 24 h were centrifuged at 800 rpm for 10 min prior to fumonisin extractions, to get rid of the excess water.

### Statistical analysis

STATISTICA 13 software was used to perform statistical analyses. To determine the effect of temperature on fumonisin production over 10 weeks, factorial analysis of variance (ANOVA), was performed. Tests for equal variance failed, due to the high amount of zero values. Logarithmic (Ln) transformations were used as follows to transform data closer to normality: Ln(x + 1), where x is the fumonisin concentration measured. Bonferroni method was used for *post hoc* multiple comparisons between time intervals. Significance was assigned to p-values <0.05.

## Results

### Different toxigenic genera

#### Aspergillus

Only AFB_1_ and AFB_2_ (Table 3) were detected in the samples and no AFG_1_ and AFG_2_. One *A. oryzae* isolate produced more than 60 mg/L AFB_1_ and more than 20 mg/L AFB_2,_ which were the highest aflatoxin levels detected in this study. *Aspergillus flavus* MRC3951 produced both AFB_1_ and AFB_2_ on the feed. Seven out of the nine *A. flavus* isolates from this study produced aflatoxins, ranging from 0.3 to 45.7 mg/L AFB_1_ and from 0.02 to 8.8 mg/L AFB_2._ Other aflatoxin-producing species from this study included *A. effusus* and *A. tennesseensis*.

**Table 3.** *Measured aflatoxin concentration (mg/L) produced by Aspergillus* spp. inoculated on abalone feed.

Isolate #	Species name	AFB_1_	AFB_2_	Total Aflatoxins
*MRL172*	*A. bridgeri*	-	-	-
*MRL292*	*A. flavus*	-	-	-
*MRL295*	*A. flavus*	*4.2851*	*0.5763*	*4.8614*
*MRL299*	*A. clavatus*	-	-	-
*MRL301*	*A. niger*	-	-	-
*MRL302*	*A. clavatus*	-	-	-
*MRL303*	*A. clavatus*	-	-	-
*MRL304*	*A. oryzae*	*60.6007*	*29.2181*	*89.8188*
*MRL309*	*A. effusus*	*0.4810*	*0.0126*	*0.4936*
*MRL332*	*A. flavus*	*45.6809*	*8.8260*	*54.5069*
*MRL341*	*A. flavus*	-	-	-
*MRL342*	*A. tamarri*	*0.3362*	*0.0096*	*0.3458*
*MRL343*	*A. tamarri*	*1.1261*	*0.2043*	*1.3304*
*MRL344*	*A. effusus*	-	-	-
*MRL358*	*A. effusus*	-	-	-
*MRL359*	*A. tamarri*	-	-	-
*MRL361*	*A. tamarii*	-	-	-
*MRL362*	*A. effusus*	-	-	-
*MRL363*	*A. flavus*	*34.1191*	*4.0938*	*38.2129*
*MRL364*	*A. effusus*	-	-	-
*MRL365*	*A. effusus*	-	-	-
*MRL366*	*A. tamari*	-	-	-
*MRL367*	*A. flavus*	*20.5714*	*2.9474*	*23.5188*
*MRL368*	*A. amoenus*	-	-	-
*MRL380*	*A. tennesseensis*	-	-	-
*MRL386*	*A. flavus*	*3.7456*	*0.1037*	*3.8493*
*MRL387*	*A. flavus*	*1.9922*	*0.0258*	*2.0180*
*MRL388*	*A. flavus*	*1.1052*	-	*1.1052*
*MRL389*	*A. effusus*	*0.3780*	*0.0777*	*0.4557*
*MRL390*	*A. effusus*	-	-	-
*MRL392*	*A. tamarii*	-	-	-
*MRL396*	*A. oryzae*	-	-	-
*MRL410*	*A. effusus*	-	-	-
*MRL414*	*A. amoenus*	-	-	-
*MRL416*	*A. tennesseensis*	*0.7873*	*0.1352*	*0.9225*
*MRL417*	*A. cristatum*	-	-	-
*MRC3951(Positive control)*	*A. flavus*	*25.7582*	*7.9090*	*33.6672*
*Sterile control*		*0.3655*	*0.0534*	*0.4189*
*Natural control*		-	-	-

#### Penicillium

Ochratoxin A (OTA) was not detected in any of the feed samples inoculated with *Penicillium* isolates in this study. No OTA was detected in either the sterile control or the natural control. *Penicillium viridicatum* MRC356 produced OTA as high as 536.4 mg/L on abalone feed. However, this value exceeded the highest measurable concentration, and could therefore be regarded as an inaccurate value, but is still regarded as positive. Isolates used in this study included a number of species, namely *P. crustosum, P. chrysogenum, P. polonicum, P. melanoconidium, P. aethiopicum, P. griseofulvum, P. novae-zeelandiae* and *P. corylophilum*. These isolates were unable to produce OTA when colonising abalone feed, in contrast to the strains used as a positive control, *P. viridicatum* MRC356, which were able to produce high levels of OTA.

#### Fusarium

*Fusarium verticillioides* MRC826 along with 15 isolates in this study produced FB_1,_ FB_2_ and FB_3_ when growing on abalone feed (Table 4). No trichothecenes (TCT) were detected in any of the isolates. Feed (sterile control) used in this experiment showed low levels of natural fumonisin contamination with no TCT’s present (Table 4). All *F. verticillioides* isolates produced all three groups of fumonisins, with isolate MRL124 producing the highest concentrations. Only some of the *F. subglutinans* isolates produced low levels of fumonisins. Single isolates of *F. chlamydosporum* and *F. oxysporum* produced low levels and no fumonisins, respectively.

**Table 4.** Measured fumonisin concentration (mg/L) produced by *Fusarium* spp. inoculated on abalone feed.

Isolate #	Species name	FB_1_	FB_2_	FB_3_	Total Fumonisins
MRL115	*F. verticillioides*	0.2961	0.0261	0.0130	0.3353
MRL117	*F. verticillioides*	4.4801	0.6350	1.1401	6.2552
MRL118	*F. verticillioides*	3.1640	0.3072	0.3073	3.7785
MRL124	*F. verticillioides*	8.8747	1.4463	2.1139	12.434
MRL129	*F. verticillioides*	3.9765	0.2536	0.6052	4.8353
MRL253	*F. oxysporum*	–	–	–	–
MRL300	*F. verticillioides*	1.3383	0.1806	0.1579	1.6768
MRL310	*F. subglutinans*	0.0050	0.0019	0.0013	0.0082
MRL311	*F. subglutinans*	0.0017	0.0013	0.0006	0.0036
MRL312	*F. subglutinans*	0.0070	0.0036	0.0037	0.0143
MRL313	*F. subglutinans*	–	–	–	–
MRL336	*F. verticillioides*	5.5711	0.9296	0.6713	7.1720
MRL353	*F. subglutinans*	0.0002	0.0003	–	0.0005
MRL354	*F. subglutinans*	0.0005	0.0002	–	0.0007
MRL369	*F. subglutinans*	–	–	–	–
MRL383	*F. subglutinans*	–	–	–	–
MRL384	*F. subglutinans*	–	–	–	–
MRL385	*F. verticillioides*	2.2452	0.3131	0.1504	2.7087
MRL393	*F. chlamydosporum*	0.0070	0.0015	0.0017	0.0102
MRL394	*F. verticillioides*	0.5731	0.0646	0.0388	0.6765
MRL421	*F. verticillioides*	0.6911	0.0560	0.0152	0.7623
MRC826(Positive control)	*F. verticillioides*	1.0344	0.1466	0.0130	1.194
Sterile control	–	0.0020	0.0005	0.0002	0.0027
Natural control	–	–	–	0.0003	0.0003

### Fumonisin production at different temperatures

**Figure 1.**
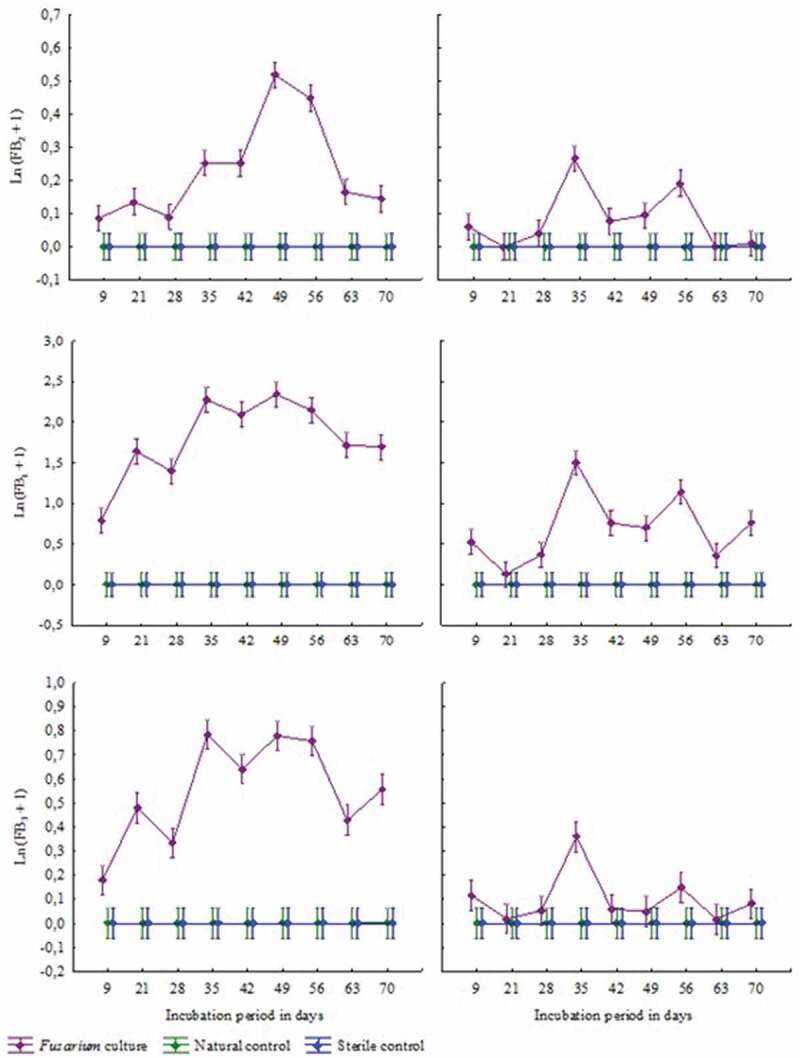
Fumonisin production on abalone feed over 10 weeks at 16°C (left) and 26°C (right), top to bottom FB1, FB2, and FB3.

Over the 70-day incubation period fungal growth could be seen at both incubation temperatures on feed inoculated with *Fusarium verticillioides*, and natural controls, while no fungal growth took place on sterile control samples. No fumonisins were detected in the sterile controls. Although growth was visible on the natural controls from as early as 9 days, no fumonisins were detected in any of these samples (Figure 1). Fumonisin levels stayed stable in both the sterile and natural controls over 70 days. In contrast, fumonisin production could be measured in samples inoculated with *F. verticillioides* from day 9. A significant increase in production of FB1, FB2 and FB3 took place at day 35 for both the incubation temperatures (Figure 1). The highest total fumonisin levels were measured at 16°C after 49 days (Table 5). The measured concentration was 11.7533 mg/L while the highest levels at 26°C were 4.3225 mg/L, measured after 35 days (Table 5). Overall, fumonisin production was higher at 16°C than at 26°C. Fumonisin B1 was the most abundantly produced fumonisin at both temperatures by the *Fusarium* isolate.

**Table 5.** Fumonisin production on abalone feed, by *Fusarium verticillioides*, at 16°C and 26°C, over a 70 day incubation period.

Incubation period (days)	FB_1_ (mg/L)		FB_2_ (mg/L)		FB_3_ (mg/L)		Total Fumonisins (mg/L)	
	16°C	26°C	16°C	26°C	16°C	26°C	16°C	26°C
9	1.2298	0.7708	0.0901	0.0632	0.1983	0.1258	1.5182	0.9598
21	4.1431	0.1386	0.1458	0.0000	0.6185	0.0187	4.9074	0.1573
28	3.0812	0.4684	0.0947	0.0408	0.3994	0.0541	3.5753	0.5633
35	8.8548	3.5762	0.2886	0.3064	1.1970	0.4399	10.3404	4.3225
42	7.2194	1.1939	0.2862	0.0802	0.9019	0.0613	8.4075	1.3354
49	9.8630	1.0196	0.6909	0.0996	1.1994	0.0509	11.7533	1.1701
56	7.7537	2.2176	0.5677	0.2117	1.1606	0.1617	9.4820	2.5910
63	4.6020	0.4415	0.1816	0.0000	0.5444	0.0176	5.3280	0.4591
70	4.5805	1.1579	0.1556	0.0108	0.7579	0.0850	5.4940	1.2537

### Water exposure

**Figure 2.**
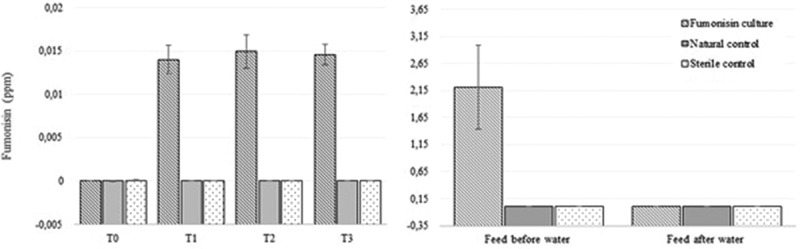
Total fumonisin concentrations in water (left) measured at four different times and in abalone feed (right), measured before water exposure and after 24 h of exposure to autoclaved seawater.

Results showed a significant increase in total fumonisins measured in the water at the first measurement taken after contaminated feed was placed in the water and a significant decrease in fumonisin concentrations in feed (no fumonisins detected) that was soaked in seawater for 24 h (Figure 2).

## Discussion

South African abalone farms are located along the coastline where the climate is known to be humid, creating the optimum conditions for fungal growth when compound feeds are stored (Whitlow & Hagler Jr. 2005). Some fungal genera such as *Fusarium, Aspergillus* and *Penicillium* produce toxic secondary metabolites better known as mycotoxins (Bezuidenhout et al. 1988; Cole 1986; Wei and Jong 1986; Gelderblom et al. 1988; Cutuli et al. 1991; Rheeder et al. 1992; Cabañes et al. 2010). Mycotoxin production is species- and strain-dependent, and is influenced by the growth substrate and environmental conditions (D’Mello and Macdonald 1997; Melcion et al. 1997; Brzonkalik et al. 2011; Garcia et al. 2012).

The first part of this study aimed to assess the potential of fungal isolates to produce mycotoxins when re-inoculated onto abalone feed. Isolates used during this study were previously isolated from abalone feed and feed ingredients (Greeff-Laubscher et al. 2018). In total 94 isolates were inoculated onto abalone feed to assess potential mycotoxin production at standardised temperatures over 6 weeks. Formulated abalone feeds are made up of a mixture of grains together with protein, making it an ideal substrate, not only for fungal growth but also for mycotoxin production (Diener and Davis 1969; Burgess et al. 1981; Cassini 1981; Greeff-Laubscher et al. 2018). This was indeed the finding for the positive control strains in this study. All three strains that were used as positive controls from each genus were able to produce their respective mycotoxins.

Most *Aspergillus flavus* strains are known aflatoxin producers (D’Mello and Macdonald 1997; Dutta and Das 2001; Moss 2002a; Bennett and Klich 2003; Johnson et al. 2004; Manning 2005; Klich 2007). Even though all the isolates in this study were subjected to the same environmental conditions during incubation, two *A. flavus* isolates did not produce aflatoxins while the others produced varied concentrations of aflatoxins, with the highest total aflatoxin level of 54.5 mg/L (MRL332) detected on abalone feed. This is relatively low compared to previous studies where aflatoxin production ranged from less than 10 mg/L to >100 mg/L in sucrose based liquid growth medium (Horn and Dorner 1999; Geiser et al. 2000). Even though *A. flavus* and *A. oryzae* have several homologues of aflatoxin biosynthesis pathway genes, genetic defects have led to the silencing of the aflatoxin pathway in *A. oryzae* (Watson et al. 1999; Takahashi et al. 2002). This has been supported by many studies (Wei and Jong 1986; Barbesgaard et al. 1992; Geiser et al. 2000; Bennett and Klich 2003; Klich 2007). Blumenthal (2004) lists a few other toxic secondary metabolites produced by *A. oryzae*, but supports the theory that *A. oryzae* does not produce aflatoxins (Geiser et al. 2000). In contrast, two different studies found that *A. oryzae* originally isolated from wheat grains and meat products, produced aflatoxins when re-inoculated onto wheat and liquid media respectively (El-Kady et al. 1994; Atalla et al. 2003). Despite these findings, the overall agreement is still that *A. oryzae* is non-toxigenic, and that isolates could have been wrongly identified, such as *A. oryzae* NRRL1988, which was reported to produce aflatoxins but was later re-identified as *A. parasiticus* (Fennell and Morse 1976; Blumenthal 2004). This possibility is not unlikely as *A. oryzae* falls within the *A. flavi* clade, and is closely related to toxigenic species (Samson et al. 2014). Considering the effect that the growth substrate and other environmental conditions have on mycotoxin production (D’Mello and Macdonald 1997), we suggest that some *A. oryzae* isolates are toxigenic but that they are dependent on the substrate for mycotoxin formation. From this study, one of the two *A. oryzae* isolates tested produced both AFB1 and AFB2. Aflatoxin production by *A. oryzae* MRL304 was exceptionally high, the highest of all the isolates tested. Thus it is suggested that more research is conducted on this specific isolate to confirm identification and toxin production on a variety of growth substrates.

No OTA was detected in abalone feed inoculated and incubated with *Penicillium* species. Isolates used in this study were unable to produce detectable limits of OTA when colonising abalone feed, in contrast to the strain used as a positive control, *P. viridicatum* MRC356, which produced high levels of OTA. However, *P. viridicatum* MRC356 produced OTA concentrations exceeding the maximum level of detection. This is an indication that if toxigenic *Penicillium* species colonise abalone feed, ochratoxins can be produced – but because the isolates tested in this study did not produce detectable levels of OTA, they are potentially non-toxigenic and therefore not a concern to the abalone industry.

*Fusarium verticillioides* is a species well known to produce fumonisins on many different substrates (Bezuidenhout et al. 1988; Marasas et al. 1988; Rheeder et al. 1992; Beukes 2015; Janse van Rensburg et al. 2017). All the *F. verticillioides* strains in this study produced mycotoxins, ranging from 0.33 mg/L to 12.43 mg/L. The strains with the highest levels were almost 10 times higher than the positive control. The isolate used as a positive control, *F. verticillioides* MRC826, produced surprisingly low levels of fumonisins on abalone feed, with a total fumonisin concentration of only 1.19 mg/L, of which 1.03 mg/L were FB1. This is contrary to a study by Gelderblom et al. (1988) who inoculated the same strain (*F. verticillioides* MRC826) onto maize and incubated at the same temperature for a shorter period of time. They were able to extract up to 2 g FB1 from 1 kg culture material (2000 mg/L), indicating that although formulated abalone feed favours fumonisin production, it may not be the best substrate to promote fumonisin production specifically for *F. verticillioides* MRC826. However, the higher production levels of the other *F. verticillioides* isolates tested in this study indicate that compound abalone feed is a suitable substrate for fumonisin production, and that their ability varies between strains. Fumonisin B1 was the most abundantly produced toxin throughout this study. Formulated abalone feed and shrimp feed consists of similar ingredients, thus it would be expected that isolates from these two feeds would perform similarly in terms of mycotoxin productions (Anukul et al. 2014). However, FB1 production by isolates in this study ranged from similar to higher levels than those reported for *F. verticillioides* strains isolated from shrimp feed (Anukul et al. 2014). Isolates from that study (Anukul et al. 2014) produced FB1 ranging between 1600 and 3600 ng/g (1.6–3.6 mg/L). The difference in results could be because toxin production of isolates from Anukul et al. (2014) were tested on sterile maize and not formulated feed, which is therefore not a true reflection of the anticipated FB1 production when growing on formulated feed. Furthermore, even though incubation temperatures for the two studies were similar, incubation time in the current study was 4 weeks longer, giving the fungal isolates more time to produce mycotoxins (D’Mello and Macdonald 1997).

Many authors have stated that fumonisin production is influenced by environmental factors such as substrate, pH, temperature and humidity (D’Mello and Macdonald 1997; Melcion et al. 1997; Parsons 2008; Garcia et al. 2012; Janse van Rensburg et al. 2017). In the first part of this study, it was concluded that *F. verticillioides* isolates, originally isolated from abalone feed, can produce fumonisins when re-inoculated onto abalone feed and left to colonise. This should be of concern, because abalone farms in South Africa are mainly located along the coast where humidity is high with average temperatures of 16°C ± 2°C and 26°C ± 2°C during winter and summer months, respectively. These temperatures fall well within the range required for fungal growth and fumonisin production by *Fusarium* species (Alberts et al. 1990; Janse van Rensburg et al. 2017).

The second part of the current study evaluated fumonisin production by *F. verticillioides*, isolated from abalone feed, on the feed at two different temperatures, over a period of 10 weeks. Conditions in this study simulated storage temperatures of abalone feed on abalone farms in South Africa. This fungus was able to grow at both temperatures without difference in macro-growth characteristics, except for a slight colour change from white to beige. These results support the findings of Cahagnier et al. (1995), that fumonisin production can be influenced by environmental factors without influencing the fungal growth. Therefore, when growth is visible, regardless of the amount of biomass, it is possible that fumonisin production is in progress, but mycotoxin analyses is needed to confirm the level of contamination (Melcion et al. 1997). Although previous studies reported 25°C ± 2°C to be optimal temperatures for fumonisin production (Alberts et al. 1990; Melcion et al. 1997), this study showed that fumonisin production was higher at 16°C than at 26°C (Figure 1). However, the fumonisin production per unit in this study was not even a fraction of the amount reported by previous studies, where fumonisin production on maize measured >14,000 mg/L and 850 mg/L, after 7 and 10 days respectively (Alberts et al. 1990; Melcion et al. 1997). In contrast, Garcia et al. (2012) reported less than 0.02 mg/L fumonisin after *F. verticillioides* was incubated on sterile soybeans for 21 days at different temperatures ranging between 15°C and 30°C. Although the fumonisin levels measured in the latter study were lower than the levels measured in our study, it is closer to this study and more comparable. One of the differences between these studies is the growth substrate, which is categorised as a biological factor that influences mycotoxin production. There appears to be a link between the protein content of these substrates and the fumonisin production reported by the different authors, including the results found in this study. Soybeans have the highest protein content of 48–50%, but low levels of fumonisin production were reported on this substrate (Garcia et al. 1997, 2012). Abalone feed used in this study contains 35% protein, and fumonisin levels were measured between 0.157 and 11.753 mg/L, while maize contains only 8–11% protein, but had the highest fumonisin production reported (Alberts et al. 1990; Melcion et al. 1997; South African Grain Laboratory 2011; FAO 2016; Marifeed 2016). Although more research is needed, it could be hypothesised that protein content in the growth substrate influences the fumonisin production, and that higher protein content reduces fumonisin production. This could possibly be because of the difference in solubility of soybean protein compared to maize protein (Castro-Rubio et al. 2006). Therefore, the amount and source of protein in abalone feed should be taken into consideration when formulating feed. This is especially important as agricultural commodities used in processing can also be a source for fumonisin contamination (Greeff-Laubscher et al. 2018).

In contrast to the *Penicillium* isolates from this study that appear to be non-toxigenic, results showed that some of the *Fusarium* and *Aspergillus* isolates are toxigenic and can produce potent mycotoxins when grown on compound abalone feed under certain conditions. Although *Aspergillus* spp. isolated from abalone feed were able to produce aflatoxins when re-inoculated onto abalone feed, no detectable levels of aflatoxins were measured on abalone feed and ingredients in a previous study (Greeff-Laubscher et al. 2018). Therefore, when feed is kept fresh and stored correctly it lowers the risk of aflatoxin contamination. This was confirmed by the absence of detectable toxins in the natural control samples. These samples were not inoculated with a pure culture but rather left to mould naturally, in order to reflect feed that could be left in storerooms to mould.

With more species present and representing many different genera, interactions between species, such as competition for nutrients, takes place. When naturally-occurring fungal species colonised the feed it was likely that either none of the mycotoxins tested in this study were produced above detectable limits; or it is possible that these interactions had a detoxifying effect on the feed, as mycotoxin concentrations measured on sterile feed were higher than on the natural controls. Mycotoxin production in this study was shown to be strain-dependent, thus it is recommended that the industry make use of routine screening for possible toxigenic fungal contamination in parallel with mycotoxin contamination on feed. To date, mycotoxin risk assessments to establish allowable mycotoxin contamination levels in abalone feed and ingredients, have not been conducted for the South African market. It is expected that high levels of mycotoxins can decrease the growth rate in abalone, similar to other aquatic animals. However, fumonisin levels measured in this study were substantially lower than any current international maximum allowable limits (Food standards agency; FDA 2001; National Department of Agriculture 2006). Abalone feed used in this study had a moisture content of 8.5–11.5%.

The abundant FB1 production (Figures 1 and 2) is in agreement with current available literature (Schumacher et al. 1995; Melcion et al. 1997; Rheeder et al. 2002; Moss 2002b; Waśkiewicz et al. 2015). While a number of studies investigated the effect of fumonisins on aquatic animals such as catfish and Nile tilapia (Goel et al. 1994; Tuan et al. 2003; Manning 2005; Soriano et al. 2005), this is the first study to investigate the effect of seawater on fumonisin-contaminated abalone feed, and showed that FB1 was the only fumonisin that could be detected in the water even after 24 h. This is especially important in understanding the risks involved when feed is not consumed immediately. During this study, fumonisin levels in the abalone feed decreased significantly when submerged into the water, while FB1 concentration in the water increased significantly (Figure 2). This is a strong indication that fumonisins leach from the feed into water, in less than 2 h. Although this reduces the concern of abalone consuming fumonisin-contaminated feed, it increases the potential risk to the direct surrounding environment. If fumonisin-contaminated water gets in contact with soil at any stage, fumonisins could stay behind posing a threat to the surrounding ecosystem (Williams et al. 2003). It is worth noting that the fumonisin concentrations measured in the water were below 1 mg/L due to the high dilution factor. The same feed to water ratio used on abalone farms were used in this study. Considering the high dilution factor, the environmental risk is considered to be exceedingly small and unlikely to be problematic. However, it was previously suggested that more studies should be conducted on a wider scale to provide detailed explanation of fumonisin migration in the environment and the risk for the ecosystem and wild fish in their natural habitat (Waśkiewicz et al. 2015). Therefore, it is recommended that the presence of fumonisins and other mycotoxins be monitored, not only on abalone farms but on any animal farm that uses compound feed, until the effect that migrating fumonisins or any mycotoxins have on the environment and wild fish, is fully understood and proven to have no damaging effect.
